# Removal of Nutrients from Water Using Biosurfactant Micellar-Enhanced Ultrafiltration

**DOI:** 10.3390/molecules28041559

**Published:** 2023-02-06

**Authors:** Sarjana Binte Rafiq Era, Catherine N. Mulligan

**Affiliations:** Department of Building, Civil and Environmental Engineering, Concordia University, Montreal, QC H3G 1M8, Canada

**Keywords:** micellar-enhanced ultrafiltration (MEUF), nutrients, biosurfactant, sophorolipid

## Abstract

The removal of NH_4_^+^, NO_3_^−^, and NH_3_^−^ from wastewater can be difficult and expensive. Through physical, chemical, and biological processes, metals and nutrients can be extracted from wastewater. Very few scientific investigations have employed surfactants with high biodegradability, low toxicity, and suitability for ion removal from wastewater at different pH and salinity levels. This research employed a highly biodegradable biosurfactant generated from yeast (sophorolipid) through micellar-enhanced ultrafiltration (MEUF). MEUF improves nutrient removal efficiency and reduces costs by using less pressure than reverse osmosis (RO) and nanofiltration (NF). The biosurfactant can be recovered after the removal of nutrient- and ion-containing micelles from the filtration membrane. During the experiment, numerous variables, including temperature, pH, biosurfactant concentration, pollutant ions, etc., were evaluated. The highest amount of PO_4_^3−^ was eliminated at a pH of 6.0, which was reported at 94.9%. Maximum NO_3_^−^ removal occurred at 45.0 °C (96.9%), while maximum NH_4_^+^ removal occurred at 25.0 mg/L (94.5%). Increasing TMP to 200 kPa produced the maximum membrane flow of 226 L/h/m^2^. The concentrations of the contaminating ion and sophorolipid were insignificant in the permeate, demonstrating the high potential of this approach.

## 1. Introduction

Globally, nutrient contamination is one of the most significant threats to aquatic ecosystems [1,2] There is uncertainty at every stage of the process, from the generation of pollutants to their final ecological and economic effects. Excessive nutrient loading endangers aquatic ecosystems by changing aquatic biodiversity and biogeochemical processes [2,3]. The bioaccumulation caused by organic inputs and agricultural runoff threatens the world’s freshwater streams [4]. Despite the installation of strict environmental laws to address human impacts on aquatic species, the effects of nutrient loading on the functioning of stream ecosystems remain unknown [1,2,3].

It has proven difficult to remove different nutrients (such as phosphorus, nitrate, and ammonium) from wastewater [4,5]. Nitrogen and phosphorus are the two most important nutrients to take into consideration when talking about the discharges of treated wastewater [3,4]. They persist in streams that have been biologically treated, demanding additional sophisticated treatment. It has been demonstrated that the release of nitrogen and phosphorus accelerates lake eutrophication and increases algal bloom and freshwater habitats rooted in shallow streams [5,6]. The use of water containing algae and aquatic plants for drinking water, fish culture, or recreation produces various difficulties, including dissolved oxygen depletion in water bodies, toxicity to aquatic life, and a decrease in the efficiency of chlorine disinfection. Moreover, nitrates, the nitrogen byproducts of nitrification, are notorious for their lethal effects on infants. Septic systems, animal feedlots, agricultural fertilizers, manure, industrial wastewater, sanitary landfills, and rubbish dumps are all common causes of excess nitrate reaching lakes and streams [4,5,6].

In aquatic environments, ammonia creates nitrogenous oxygen demand, eutrophication, and changes in fish health. Nitrification is the cause of biological oxygen demand (NBOD) due to nitrogen. In the process of nitrification, dissolved oxygen (O_2_) is used to react with NH_3_. As a result, species have a limited availability of O_2_. As in terrestrial situations, nitrification can cause eutrophication by producing nitrate [6,7,8,9]. Nitrophilous algae and macrophytes may form large blooms in standing water [6,7]. This strains resources and can potentially indirectly harm species via the formation of algae [8,9]. Ammonia, on the other hand, can directly impact animals through skin absorption. Exposure to ammonia has been linked to fish mortality as well as alterations in fish development, gill condition, organ weights, and red blood cell concentrations [9,10].

Numerous studies have been conducted on the physical and chemical removal of metals and nutrients from wastewater [11,12]. Additionally, research has been conducted on the removal of metals and nutrients from wastewater using a synthetic surfactant. However, relatively few research studies have been conducted on the removal of contaminants via biotreatment, such as biosurfactants. In terms of environmental sustainability, biodegradability, and eco-friendliness, micellar-enhanced ultrafiltration (MEUF) with biosurfactants surpasses most other known treatment methods [13,14,15,16]. MEUF is a straightforward filtration method. The biosurfactant forms micelles in the feed solution that react with the waste molecule and the biosurfactant/contaminant complex is ultimately removed from the filtrate by the ultrafiltration process [11,13]. The MEUF based on surfactants has been evaluated to separate multivalent anions and cations [14]. It can operate well at high temperatures, which is essential for treatment processes.

This study was conducted to investigate the effectiveness of sophorolipids in removing nutrients (ammonia, phosphate, and nitrate) from water. For this investigation, a sophorolipid was utilized as a biosurfactant in the micellar-enhanced ultrafiltration (MEUF) system. This allowed for the elimination of the nutrients. The optimal conditions for the reduction of ammonia, phosphate, and nitrate were examined, which included the pH level, initial concentration of anions, sophorolipid concentration, temperature, and transmembrane pressure. When applied above its critical micellar concentration and in a reasonable ratio with the pollutant ion, sophorolipids help the MEUF system to remove ammonia, phosphate, and nitrate.

**Figure 1:**
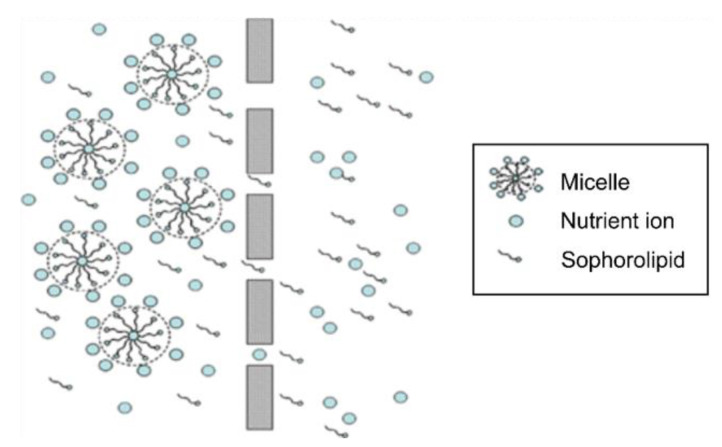
Schematic diagram of micellar-enhanced ultrafiltration.

MEUF is a membrane-based approach to removing metal ions, organic pollutants, and inorganic chemicals from water supplies. In this method, surfactants are introduced into the aqueous stream at concentrations that are either comparable to or higher than their respective critical micelle concentrations (CMCs). The lowest micellar concentration at which micellization may occur is referred to as the crucial micellar concentration (CMC). At this surfactant concentration, the surfactant monomers will begin to combine and form aggregates that are referred to as micelles [14,15]. During the MEUF procedure, cations are bound to the negatively charged micelles. The membrane rejects cations and anions with larger molecular sizes than the pores. More specific to our study, the ions NH_4_^+^, PO_4_^2−^, and NO_3_^−^ were bound to parts of the sophorolipid that were hydrophilic. Because the aggregates were larger than the pore diameters of the hollow-fiber membrane filter, they were unable to pass through the membrane. On the other hand, clean water with a small quantity of the sophorolipid and ions (NH_4_^+^, PO_4_^3−^, NO_3_^−^) did flow through the membrane. In this part of the investigation, the performance of the MEUF system was investigated according to a variety of operating parameters, including transmembrane pressure (TMP), temperature, fouling, and sophorolipid concentration. In addition to this, the activity of the sophorolipid was observed while ions were present (NH_4_^+^, PO_4_^3−^, NO_3_^−^) [1,2]. Figure 1 shows that micellar-enhanced ultrafiltration (MEUF) is a combination of two technologies, where in the first step the formation of micelle by the supply of biosurfactant starts, and then micelles are removed through ultrafiltration at a suitable concentration higher than the CMC through the ultrafiltration membrane.

The dissolution of metal ions and organic molecules in micelles can be attributed to the action of electrostatic or Van der Waals forces. After this, the micelle solution is passed over an ultrafiltration membrane that has a suitable molecular weight cut-off (MWCO) size. As a result, the micelles that are associated with soluble pollutants can be removed by the ultrafiltration membrane [1,4,14]. In a general sense, the retention coefficient of the pollutant that is being eliminated rises when the surfactant concentration in MEUF rises higher than the CMC [1,2,14]. MEUF possesses a variety of benefits, some of which include low operating costs, a high removal efficiency, and a high permeate volume flux, to mention a few of these benefits. In a nutshell, this system combines the high selectivity offered by reverse osmosis with the high flux offered by ultrafiltration. Because of these characteristics, MEUF is applied in the process of removing heavy metals [1,2,7,16].

MEUF offers numerous advantages, such as low operating costs, high removal efficiency, and a large permeate volume flux. This method combines the high flux of ultrafiltration with the superior selectivity of reverse osmosis. MEUF is used to remove both anions and cations because of these properties. However, one of the system’s major shortcomings is the decrease in permeate flux caused by various experimental conditions, including membrane fouling [1,15,16,17,18,19,20], which can be minimized by regular membrane cleaning and maintenance. The objectives of this research are to evaluate the effectiveness of MEUF with a sophorolipid biosurfactant for removing nutrients of various forms of nitrogen (ammonium and nitrate) and phosphate from water [1,17,18].

## 2. Results

### 2.1. CMC Determination

The critical micelle concentration (CMC) of the sophorolipid used in this study was determined to be 30 mg/L or 0.003% of the sophorolipid. By determining the CMC of the biosurfactants, the minimum concentration at which micelles will form and the lowest concentration at which biosurfactant solutions will operate optimally are calculated. In addition, by measuring the CMC of the experiment’s effluent, the biosurfactant concentration in the effluent may be calculated, as well as the relationship between biosurfactant adsorption to the media and biosurfactant concentration. There is a substantial variance in the CMC values of several types of biosurfactants. The lower the CMC value, the less biosurfactant will be required [1,2,17,18,19,20]. The cost of biosurfactants constitutes a considerable component of the total cost of remediation, proportional to the amount of biosurfactant employed. Therefore, the ideal characteristic of a biosurfactant is a low critical micelle concentration (CMC) [18,19,20,21,22]. At that concentration, the measured surface tension was 44 mN/m. Finding the intersection of two tangent lines was used to establish this CMC [20,21].

### 2.2. Effect of pH on Removal Rate

The proposed pH range for this experiment was between 6.0 and 10.0. At a lower pH, the rate of nutrient ion elimination is greater. Based on the pH of the sophorolipid and other chemical characteristics of the pollutant anion and cation, the pH was determined.

**Figure 2:**
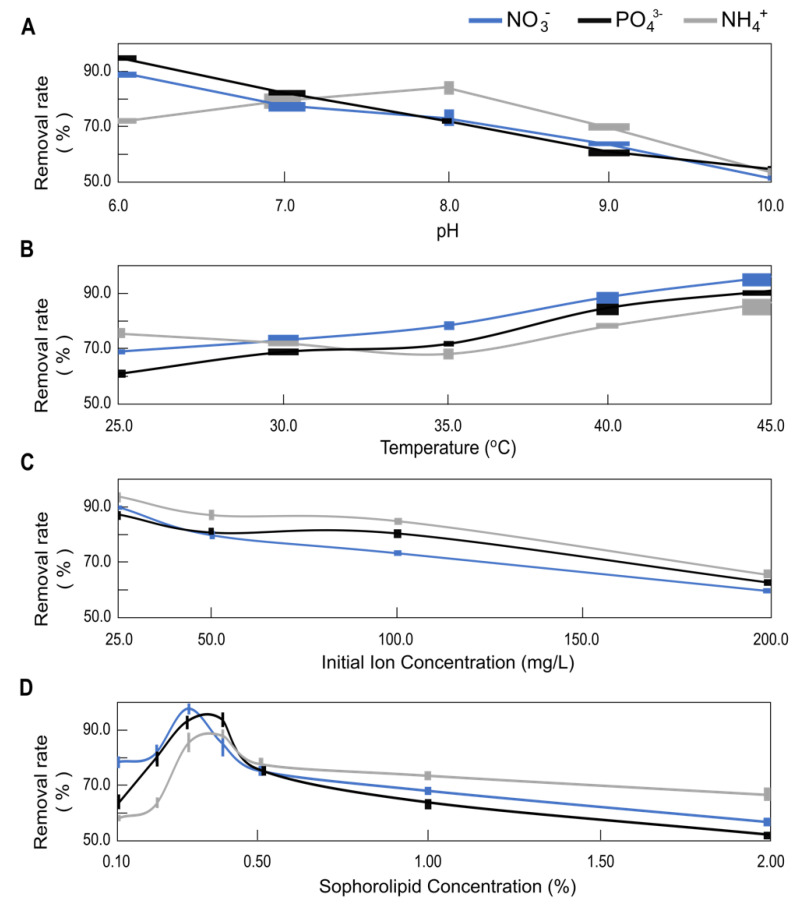
Effect of (**A**) pH, (**B**) temperature, (**C**) initial ion concentration, and (**D**) sophorolipid concentration on the removal rate of NO_3_^−^, PO_4_^3−^, and NH_4_^+^.

The sophorolipids used in the study are acidic with a pH of 4.5 and sensitive to pH, temperature, and electric fields. This may result in alterations to their performance [1,19,22,23]. By adjusting the pH, several sophorolipid aggregate states may be produced. At neutral pH values, sophorolipids exhibit strong emulsifying activity. In pure water, when pH decreases below 6.0, the emulsion’s stability increases. However, in the presence of ions, considering the pH of the sophorolipid and the pH of the employed ions, the optimal pH range was determined to be between 6.0 and 10.0. Going below 6.0 in the presence of ions would cause a gel formation on the membrane surface causing more fouling, while going beyond 10.0 the emulsification stability of SL drops to a level that would reduce the system efficiency at a significant rate. As seen in Figure 2A, at lower pH levels (6.0–8.0), nitrate, phosphate, and ammonium removal rates were desirable with values between 70% and 96%. The removal rate varies based on the ions and the overall pH of the solution. In the solution, there are two anions and one cation [1,2,23,24,25,26]. So, the impact of pH on the removal rate can be explained by anion exchange and reduction of the anions in the solution (NO_3_^−^, PO_4_^3−^) [24,25,26].

### 2.3. Effect of Temperature on Removal Rate

Based on the findings seen in Figure 2B, the removal rates of nitrate, phosphate, and ammonium improve with increasing temperature due to an increase in membrane flux [9,10,11,12,13,14,27] induced by the membrane material’s thermal expansion and decreased solution viscosity [27]. Since the CMC of the surfactant fluctuates with temperature, the temperature is the most important parameter for MEUF [28,29,30,31,32,33,34]. The effect of temperature on the process was studied in this experiment in the range of 25.0 °C to 45.0 °C. Other parameters were constant. A noticeable decrease in the flux was observed when lowering the temperature below 25.0 °C, caused by higher viscosity of the SL solutions, and above 45.0 °C, due to greater concentration polarization. This was also the case for other surfactants, e.g., CTAB, CPC, and rhamnolipid [1,35,36,37,38].

### 2.4. Effect of Concentration on Removal Rate

In Figure 2C, the removal rate at various concentrations (25.0, 50.0, 100.0, 200.0 mg/L) of initial nitrate (NO_3_^−^), phosphate (PO_4_^3−^), and ammonium (NH_4_^+^) was evaluated. This range of ion concentrations reflects values that are 100–250 times more than the proportion of actual polluting ions found in Montreal’s lake water. For this research study, ions with a small diameter for ultrafiltration were chosen based on pore size. Using a lower concentration would not have been useful for understanding the micelle formation with SL and the influence of other parameters (pH, temperature, permeate flow, ion concentration, transmembrane pressure) owing to ions lost before micellization occurs. The presence of untreated ions in permeate solutions becomes negligible when a concentration 100–250 times higher is used.

Figure 2C depicts the effect of starting concentration on the elimination rate. With an increase in the initial concentration, the removal rate decreases substantially [1]. Figure 2D illustrates the relationship between the removal rate and the changing sophorolipid (SL) concentration in the solution. In this experiment, the SL concentration was examined at 0.10%, 0.20%, 0.30%, 0.40%, 0.50%, 1.00%, and 2.00% to determine the nitrate, phosphate, and ammonium ion removal rate. The greatest NH_4_^+^ removal rate was 88.8% at 0.40% SL concentration, while the lowest rate was 58.7% at 0.10% SL concentration [1]. Initially, the experiment was carried out between CMC (0.003%) and 10 CMC of SL (0.03%) and the result was unsatisfactory with a removal rate below 40%. After increasing SL concentration from 33 × CMC (0.1%) to 133 × CMC (0.4%), the results gave better removal rates ranging between 60% and 96% due to a greater surface of attachment for the ions. In other studies, it was shown that the removal of pollutant ions and metal ions was higher when the ion-to-surfactant ratio was between 1:1 and 1:1.5, and in this case the ion-to-biosurfactant ratio was 1:1.4 (wt/wt) [1,2,4,6,7].

This indicates that the rate of nutritional ion elimination is related to SL concentration. This indicates that raising the SL content from 0.025% to 0.10% facilitates nutrient clearance. When the concentration of SL in the feed solution increases, so does the concentration of micelles, which enhances the binding of nutritional ions [1,33,34,35,39]. Table 1 summarizes the maximum and minimum values of the removal of the studied ions affected by pH, temperature, ion, and sophorolipid concentrations. Typical standard deviations on values were reported between 0.2% and 2.0% for pH, 0.5% and 2.0% for temperatures and transmembrane pressures, 1.0% for ion and SL concentrations, 1.0% and 3.0% for permeate fluxes, and finally, between 1.0% and 5.0% for removable rates.

Results indicated that 98.9% of NO_3_^−^ was removed at a concentration of 0.40% SL, with the lowest removal rate occurring at a concentration of 2.00% SL. At a pollutant-ion-to-sophorolipid ratio of 1:1.4 (wt/wt), the maximum clearance rate was reported. This proportion remained constant throughout the tests. The better removal rate at a higher concentration may be explained by the increased ratio of biosurfactant to pollutant ion, which increases the availability of the biosurfactant for the attachment of the pollutant ion. The fouling of the membrane began beyond this point due to the increased viscosity [1,35,36]. After a certain SL concentration, fouling and the increased viscosity of the solution reduced the removal rate [35,36,40,41,42]. In this instance, the clearance rate is reduced when the concentration exceeds 0.40%. The apparent tension between the feed solution and the permeate was measured to verify the sophorolipid (SL) content of the solution both before and after the test using a tensiomat. The surface tension of the feed solution with the addition of SL was reduced to 41 mN/m, which is below that of the CMC. Therefore, micelles were formed as the concentration was above the CMC [36,37,39]. The surface tension of the permeate solution increased to 65 mN/m after filtration, which was slightly lower than the surface tension of pure water (72 mN/m). As the SL in the micelles was rejected by the membrane during filtration, only a few monomers passed through the membrane [37,38].

To verify the relationship between the independent variables of pH, temperature, ion, and SL concentrations with the dependent removal rates, variances were calculated and compared within each group. The variance in each group was in the range of 55 to 255%. Nitrate ions were referred to as group 1, while phosphate and ammonium ions as groups 2 and 3, respectively. Our hypothesis was to assume that no relation exists between experimental conditions and removal rate values for each group. This hypothesis is valid for low *p* values up to 5%. For higher *p* values greater than 95%, the alternative hypothesis, that at least one group differs from the overall mean, prevails. A one-way ANOVA test of comparison for at least three different groups was used to calculate the probability of finding at least one group higher than the mean. *p*-values ranged between 98% for pH, 65% for temperature, 68% for ion concentration, and 83% for SL concentration. This shows that pH is the most likely to influence the process and is the principal parameter precisely controlling ion removal rates.

### 2.5. Effect of Temperature on Permeate Flux

**Figure 3:**
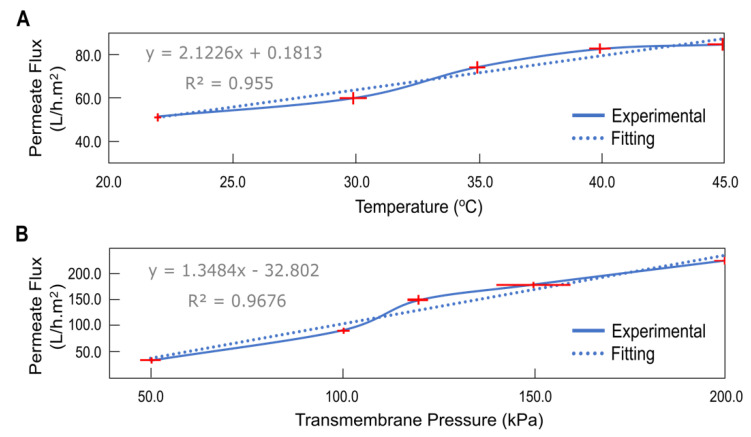
Effect of (**A**) temperature and (**B**) transmembrane pressure on permeate flux at pH 6.0 ([NH_4_^+^], [NO_3_^−^], [PO_4_^3−^] = 100.0 mg/L).

The effect of temperature on the process was studied in this experiment in the range of 22.0 °C to 45.0 °C. Other parameters were constant. The permeate flow increases as the temperature rises, owing to the thermal expansion of the membrane material and the lower viscosity of the solution. Due to the increased flow, however, more concentration polarization was observed [1,35,36,37,38]. Because the surfactant CMC varies with temperature, the temperature is the most significant parameter for MEUF. The viscosity of the synthetic solution containing the sophorolipid solution decreases as the temperature rises, causing the flux to rise. The range 22.0 °C to 45.0 °C was chosen based on the flux rate and power required to raise the temperature. Lowering the temperature below 22.0 °C would cause a decrease in the flux while going above 45.0 °C would require a considerable power supply to heat up the solution causing extra operational costs [1,38,43]. When the temperature increases, the flux also increases, as seen in Figure 3A. The viscosity of the solution containing the sophorolipid solution decreases as the temperature rises, allowing the flux to increase [38]. The flux reached a maximum of 92.1 L/h.m^2^ at 45.0 °C and a minimum of 47.6 L/h.m^2^ at 22.0 °C.

### 2.6. Effect of Transmembrane Pressure in Permeate Flux

As shown in Figure 3B, increasing the transmembrane pressure (TMP) positively influences the permeate flow, implying that as the TMP increases, the driving force starts to rise, resulting in higher flux. Furthermore, a linear relation between TMP and flux shows negligible concentration polarization [6,28]. The lowest flux was 30 L/h.m^2^ at TMP = 50 kPa, while the highest flux was 226 L/h.m^2^ at TMP = 200 kPa. Low transmembrane pressure reduces operational expenses [42,44]. Because its value was greater than the linear trendline, a second-order regression was determined as the best match, as indicated by the R^2^ value. The range between 50 and 150 kPa was chosen for the tests in which MEUF was utilized with a synthetic or biosurfactant for the removal of metal and inorganic ions [2,6,7,8]. This study was first conducted in this range and the permeate flux achieved was not satisfactory. As a result, the maximum limit of the range was increased to 200 kPa [1]. The low flux achieved at 150 kPa can be explained with the MWCO used for this experiment because with the increase in MWCO the flux significantly reduces for the MEUF process [1,2]. For the removal of ions such as nutrients, which are smaller than metal ions, the ultrafiltration cartridge of higher MWCO is necessary, which causes the flux reduction. This can be interpreted as the reason why the necessary flux was not achieved at 150 kPa. The MWCO that was used for this investigation was 10 kDa, while the MWCO that was used in the mentioned experiments that were cited ranged from 3 kDa to 5 kDa. The polarity of the two ions with differing charges, as well as the concentration of those ions, might play a part in the experiment’s permeate flux [2,6,7].

Increasing the transmembrane pressure (TMP) positively influences permeate flow, implying that as the TMP increases, the driving force starts to rise, resulting in higher flux. On the other hand, lower transmembrane pressure reduces operational costs. The range selected is 50–200 kPa since below that level there is almost no removal observed, while going above 200 kPa would significantly increase the operational power requirement, resulting in higher cost.

## 3. Discussion

The primary purpose of this study was to evaluate the effectiveness of sophorolipids (SL) in removing ammonium, phosphate, and nitrate from water. As a biosurfactant for the elimination of ammonium, phosphate, and nitrate (NH_4_^+^, PO_4_^3−^, NO_3_^−^), the SL was combined with a micellar-enhanced ultrafiltration (MEUF) system. By analyzing a variety of factors, the best conditions for each variable, including pH, initial concentration of anions, SL concentration, temperature, fouling, and transmembrane pressure, were identified. SL plays an essential role in the elimination of ions (NH_4_^+^, PO_4_^3−^, NO_3_^−^) via the MEUF system at concentrations above its critical micellar concentration [21,28,38,43,44,45,46]. The ions (NH_4_^+^, PO_4_^3−^, and NO_3_^−^) were attached to the hydrophilic parts of the SL by ion exchange with counterions for the cations or electrostatic attraction for the anions with the negatively charged sophorolipid. As the aggregates were larger than the pore diameters of the hollow-fiber membrane filter, they were unable to pass through the membrane. However, pure water with small amounts of SL and ions was able to pass through.

Based on the experimental results, the following conclusions were drawn from this investigation: The fraction of anions and cations (NH_4_^+^, PO_4_^3−^, NO_3_^−^) that were decreased was affected by variables such as pH, initial concentration, and SL concentration. A drop in pH and a rise in SL concentration greatly influenced the decrease in PO_4_^3−^ and NO_3_^−^ concentrations. Each sample had a pH of 6.0 and a concentration of 100.0 mg/L for NH_4_^+^, PO_4_^3−^, and NO_3_^−^. SL = 0.30% was selected as the best concentration for the reduction of anions. Temperature and transmembrane pressure served as critical operating parameters for the micellar-enhanced ultrafiltration system. When both were raised, the flow increased [2,43,44,45]. However, transmembrane pressure had a greater influence on the flow than temperature. The concentration of feed had no effect on the concentration of SL in the permeate. As a biosurfactant in an ultrafiltration system with micellar enhancement, SL was very effective in removing nutrients from water [1,47,48].

The high nutrient concentration in the solution, along with the high MWCO that was applied, contributed to the poor permeate flux. The decrease in flow that was caused was primarily attributable to membrane fouling being present. This effect can be mitigated by increasing the solution’s TMP and reducing the ion concentration in the solution as much as possible. When employed on a lab scale, it was difficult for MEUF to generate greater TMP. However, this issue can be resolved when applied on an industrial scale. When compared to other methods in use, MEUF features a smaller footprint and a more compact construction. Other approaches require more sludge generation and subsequent filtration to disinfect effectively [2,6,8].

In earlier research, MEUF was combined with synthetic surfactants (e.g., CTAB, CPC, ODA, DTAC) and biosurfactants (e.g., rhamnolipid) to effectively remove heavy metals (>99%) [2,6,23,24]. In another experiment, metal removal was performed in combination with sophorolipid and rhamnolipid [6], which likewise resulted in a high removal rate (>99%). Due to the ion size, competition between two differently charged particles of the ions, and the complexity of the process, there were insufficient investigations on removing nutrients from wastewater combining MEUF and biosurfactants. Only a few studies included the use of synthetic surfactants (CTAB, CPC, and ODA) for nutrient removal, and the removals ranged from 73 to 91% [23,24]. Due to the lack of research on sophorolipids with MEUF for nutrient ion removal and their effectiveness in removing heavy metals from groundwater, MEUF with sophorolipid was chosen for this research.

## 4. Materials and Methods

### 4.1. Materials

ACS-grade sodium nitrate (NaNO_3_), dipotassium phosphate (K_2_HPO_4_), and ammonium chloride (NH_4_Cl) were used and purchased from Sigma Aldrich, ON. The pH was corrected with 0.5 N nitric acid (HNO_3_) and 0.5 N sodium hydroxide (NaOH) solutions purchased from Fisher Scientific. Sophorolipid (SL) biosurfactant was produced from *Candida bombicola* cultivated on a mixture of rapeseed oil and glucose [1,2,3,4,5,8,47] and purchased from Belgium’s Ecover Company. It was composed of 30% acidic SL and 70% lactonic SL. Concentrations of 137.1 mg/L NaNO_3_, 183.4 mg/L K_2_HPO_4_, and 296.5 mg/L NH_4_Cl in double distilled water were prepared to obtain a stock solution of 100.0 mg/L NH_4_^+^, PO_4_^3−^, and NO_3_^−^. The decrease in NH_4_^+^, PO_4_^3−,^ and NO_3_^−^ by sophorolipid at various pHs and sophorolipid concentrations was studied in batch studies. To achieve equilibrium, the prepared samples were agitated at 60 rpm for 24 h, then centrifuged and analyzed.

In this work, we attempted to maximize the percentage of nutrient removal under suitable test conditions by maintaining appropriate permeate flux and maximizing the factors influencing permeate flux. For this, experiments were carried out at a flow rate of 200 mL/s by keeping the peristaltic pump at 70 rpm, the transmembrane pressure at 120 kPa with a molecular weight cut-off (MWCO) of 10,000, and an initial ion concentration of 100.0 mg/L at 22 °C, with a sophorolipid concentration of 0.30%.

### 4.2. Experimental Setup

**Figure 4:**
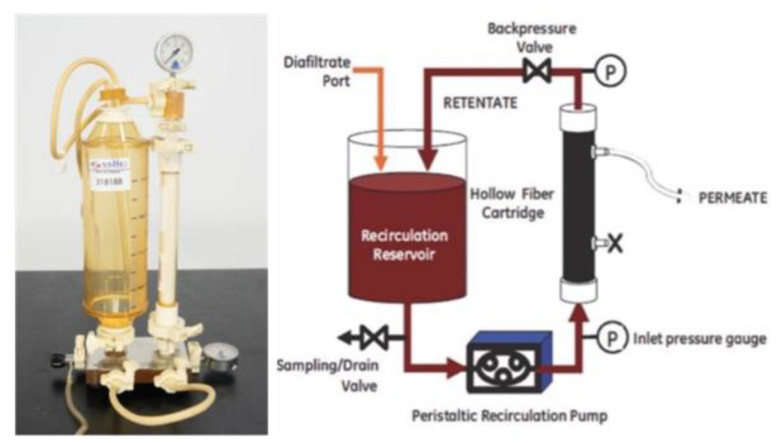
Benchtop (**left**) and schematic diagram of the experimental setup. Peristaltic pump (**right**).

The QuixStand BenchTop System (Figure 4) (M series from GE Healthcare) was used for the separation of nutrients (NH_4_^+^, PO_4_^3−^ and NO_3_^−^), which was attached to the surface of micelle from the solution of nutrient-sophorolipid. The system included a feed reservoir, peristaltic recirculation pump, inlet pressure gauge, hollow-fiber cartridge (Xampler cartridge), retentate outlet, outlet pressure gauge, sampling valve, and back pressure valve. The peristaltic pump that was included in the ultrafiltration system to pump the fluid was purchased from Watson-Marlow Company (313 S).

#### Xampler™ Cartridge

The hollow-fiber cartridge used in QuixStand BenchTop (Ultrafiltration System) was purchased from GE Healthcare. A bundle of polysulfone fibers parallels inside a plastic housing and forms the cartridge. Molecular weight cut-off (MWCO) is essential in classifying ultrafiltration membranes. The MWCO that was used in the experiments was 10,000.

### 4.3. Ultrafiltration Tests

The QuixStand BenchTop System (M series from GE Healthcare, Buckinghamshire, UK) was utilized for the separation of ions (NH_4_^+^, PO_4_^3−^, and NO_3_^−^) bound to the chromium-rhamnolipid micelles. The system comprised a feed reservoir, a peristaltic recirculation pump, an inlet pressure gauge, a hollow-fiber cartridge (Xampler cartridge), a retentate outlet, an outlet pressure gauge, a sample valve, and a back pressure valve. The Xampler cartridge was acquired from GE Healthcare. The cartridge is composed of parallel polysulfone fibers housed within a plastic container. Classifying ultrafiltration membranes is dependent on the MWCO (molecular weight cut-off). In the trials, a MWCO of 10,000 was utilized based on previous studies [1]. These tests were carried out in batches. The feed solution had a volume of 400 mL at the beginning, and the retentate stream was continually recycled. The water flux was monitored before and after the experiment at the optimal transmembrane pressure to confirm membrane fouling. It was time to clean the membrane when the water flux was less than 80–90% percent of the flux of a new membrane. The sodium nitrate, dipotassium hydrogen phosphate, and ammonium chloride salts were dissolved in distilled water to produce a stock solution of NH_4_^+^, PO_4_^3−^, and NO_3_^−^, and the required concentrations were produced by diluting the stock solution with the same water. Distilled water was used to dilute Ecover SL (41%) (SL18) to produce various molar solutions. The reservoir’s feed solution, which contained anions, cations, and SL, was fed through the ultrafiltration membrane by a peristaltic pump. The retentate solution was returned to the feed reservoir after exiting the cartridge. Ion chromatography was used to measure the concentrations of NH_4_^+^, PO_4_^3−^, and NO_3_^−^ in the permeate, retentate, and feed samples. All tests were conducted at a temperature of 22.0 °C and a pH of 6.0 unless otherwise specified. After each experiment, the flow loop was cleansed by running distilled water through the apparatus. Each test was performed three times, and the average was used to determine the outcome.

### 4.4. Analytical Methods

**Statistical method:** For each parameter, the test was run twice in duplicate, and the values were then averaged to obtain the numbers used in the table and graphs. Using ANOVA, the standard deviation (SD) values for the three sets of data were computed, and the error bars on the graph were generated using 2SD. In the first trial, the ratio of pollutant ion to sophorolipid was 13:1 (wt/wt), and the achieved removal rate was less than 85%. In the second set of experiments, the ratio of ions to sophorolipids was 1:1.4 (wt:wt). Based on the performance of the initial experimental data set, the values of the studied parameters (pH, temperature, TMP, ions, and SL concentrations) were chosen. The effect of altering a single parameter on the removal rate and permeate flux was investigated while the other parameters remained constant.

**pH:** The pH was measured using a Fisher Scientific Company AR25 Dual Channel pH/Ion Meter. Considering pH plays such a significant role in reducing NH_4_^+^, PO_4_^3−^, and NO_3_^−^ ions, the effect of varying pH levels was investigated. Because the pH of the solution after adding SL is 7.82, the solutions were tested at pH 6.0, 7.0, 8.0, 9.0, and 10.0. Each test was conducted in triplicate, and the total sample amount was 50 mL. Temperature, anion and cation concentrations, and SL concentrations were fixed at 100.0 mg/L for NH_4_^+^, PO_4_^3−^, and NO_3_^−^, respectively, and 0.30% of SL. The pH was adjusted with 0.5 N NaOH and 0.5 N HNO_3_, and the initial and final contents of NH_4_^+^, PO_4_^3−^, and NO_3_^−^ were determined using ion chromatography. Equation (1) [1,2,6] was used to calculate the percentage of anion and cation reduction:

(1) $$\begin{array}{c} \%\;〖Ion〗\_reduction\\ \;=(〖Ion\;Concentration〗\_initial\\ \;-〖Ion\;Concentration〗\_final\;)\\ \;/〖Ion\;Concentration〗\_initial\;\times \;100\% \end{array}$$

**Temperature:** A 2001 Series 8000 Resistance Temperature Detector was used for measuring temperature. The influence of temperature on removal rate was examined in this experiment by utilizing varied feed solution temperatures (25.0, 30.0, 35.0, 40.0, and 45.0 °C). Room temperature, transmembrane pressure, and pH were kept constant, and the solution had a pH of 6.0 and included 100.0 mg/L of NH_4_^+^, PO_4_^3−^, and NO_3_^−^, and 0.30% sophorolipid. Ion chromatography was used to determine the ion concentrations.

**Transmembrane pressure (TMP):** For observation of the effect of TMP on the permeate flux, various TMP values (40, 50, 100, and 150 kPa) were chosen. This experiment was performed at 22 °C and pH 6.0. The feed solution contained 0.30% SL. The permeate pressure was measured by a traceable manometer/pressure/vacuum gauge and the TMP was determined based on the following Equation (2): [1,2,6]

𝑇𝑟𝑎𝑛𝑠𝑚𝑒𝑚𝑏𝑟𝑎𝑛𝑒 𝑝𝑟𝑒𝑠𝑠𝑢𝑟𝑒 = (𝑃_𝑖𝑛𝑙𝑒𝑡_+𝑃_𝑜𝑢𝑡𝑙𝑒𝑡_)^2^ − 𝑃_𝑝𝑒𝑟𝑚𝑒𝑎𝑡𝑒_ (2)

The permeate flux was measured using Equation (3): [1,2,6]

(3) $$Flux\left(\frac{L}{m^{2}\cdot h}\right)=\left\{\frac{permeate\;flow\left(\frac{mL}{min}\right)}{cartridge\;area\left(m^{2}\right)}\right\}\times 0.06$$

The cartridge area was 140 cm^2^, and the permeate flow was measured by using the flowmeter for the permeate flow in the ultrafiltration system.

**Ion concentration:** An ion chromatograph, the 930 Compact I.C. Flex, was used to determine the starting and final ion concentrations. The ion chromatograph was connected to a sample processor in this approach, which was controlled by high-performance P.C. software (Metrohm’s MagIC Net). The software controlled and analyzed the instrument, as well as evaluated and managed the data collected in a database. The final percentage was determined by multiplying the concentrations obtained by the appropriate dilution factor. Ions in the water were identified using ion chromatography (I.C.). Each analysis required 10 mL of sample collection. For this investigation, samples of untreated wastewater and MEUF permeate and retentate were collected after 2, 5, 10, and 20 min and diluted by a factor of 10 with DI water.

**CMC determination:** The surface tension of sophorolipid in various concentrations was measured using a tensiometer according to the Du Nouy method (Fisher Scientific, Tensiomat model 21). The force required to lift a thin metal ring (platinum ring) from the solution’s surface is measured with a tensiometer. To assure the accuracy of the data, the tensiometer was first calibrated by measuring the surface tension of DI water. To measure the sophorolipid critical micelle concentration (CMC), the solution was diluted several times. [1,2,6,10] The surface tension of the solution was determined by submerging a platinum ring in the solution after each dilution step. The CMC of sophorolipid was determined using the Du Nouy method by plotting the surface tension versus biosurfactant concentration.

## 5. Conclusions

The primary purpose of this research was to devise a method for removing phosphorus, nitrate, and ammonium ions from contaminated water by using the biosurfactant with micellar-enhanced ultrafiltration technology. A sophorolipid (SL18) was used as a biosurfactant in this study for enhanced micellar ultrafiltration (MEUF) studies. The study’s goals include assessing the feasibility of employing sophorolipid SL18 to remove phosphate, nitrate, and ammonium from contaminated water and determining factors affecting efficiency. Furthermore, the parameters that influence permeate flux and removal efficiency were investigated. This study aimed to determine the best conditions for increasing permeate flux and achieving maximum efficiency. Various factors, including temperature, pH, the concentration of biosurfactants, pollutant ions, etc., were examined during the experiment. At a temperature of 45.0 °C and pH 6.0, there was a 90–96% removal rate for nitrate and phosphate. A maximum ammonium removal of 95% was achieved. These results indicate the high potential of this technique for nutrient removal.

## Figures and Tables

**Table 1:** Maximum and minimum values of the removal rates of nitrate, phosphate, and ammonium ions under different conditions of pH, temperature, and concentration.

Removal Rate (%)			
Conditions	NO_3_^−^	PO_4_^3−^	NH_4_^+^
pH = 6.0 ± 0.1	(89.6 ± 1.4)	(94.9 ± 1.0)	(71.2 ± 1.4)
pH = 8.0 ± 0.1	(72.6 ± 3.0)	(71.6 ± 1.4)	(84.1 ± 3.0)
pH = 10.0 ± 0.1	(50.2 ± 1.6)	(53.6 ± 2.5)	(52.5 ± 3.4)
T = 25.0 ± 0.1 °C	(69.1 ± 1.0)	(60.8 ± 0.9)	(75.7 ± 2.0)
T = 35.0 ± 0.1 °C	(78.7 ± 1.7)	(71.8 ± 1.0)	(68.1 ± 2.1)
T = 45.0 ± 0.9 °C	(96.9 ± 2.5)	(91.8 ± 3.1)	(87.3 ± 0.9)
IC = 25.0 ± 0.2 mg/L	(90.4 ± 1.3)	(87.8 ± 3.0)	(94.5 ± 3.5)
IC = 200.0 ± 1.8 mg/L	(59.8 ± 0.7)	(62.8 ± 2.4)	(65.7 ± 2.9)
SLC = 0.30 ± 0.01%	(98.3 ± 3.7)	(92.9 ± 3.4)	(86.3 ± 3.6)
SLC = 2.00 ± 0.02%	(56.4 ± 0.6)	(51.0 ± 1.5)	(67.0 ± 2.5)

T = Temperature; IC = Ion concentration; SLC = Sophorolipid comcentration.

## Data Availability

The data presented in this study are available on request from the corresponding author.

## References

[1] Era S.B.R. (2022). Removal of Nutrients from Water Using Biosurfactant Micellar Enhanced Ultrafiltration. Master’s Dissertation.

[2] Abbasi-Garravand E., Mulligan C.N. (2014). Using micellar-enhanced ultrafiltration and reduction techniques for removal of Cr(VI) and Cr(III) from water. Sep. Purif. Technol..

[3] Shekhar S., Sundaramanickam A., Balasubramanian T. (2015). Biosurfactant producing microbes and their potential applications: A review. Crit. Rev. Environ. Sci. Technol..

[4] Baek K., Kim B.K., Cho H.J., Yang J.W. (2003). Removal characteristics of anionic metals by micellar-enhanced ultrafiltration. J. Hazard. Mater..

[5] Batista S.B., Mounteer A.H., Amorim F.R., Tótola M.R. (2006). Isolation and characterization of biosurfactant/emulsifier-producing bacteria from petroleum-contaminated sites. Bioresour. Technol..

[6] El Zeftawy M.A.M. (2007). Use of Rhamnolipid to Remove Heavy Metals from Aqueous Streams via Micellar-Enhanced Ultrafiltration. Ph.D. Dissertation.

[7] Verma S.P., Sarkar B. (2018). Simultaneous removal of Cd (II) and p-cresol from wastewater by micellar-enhanced ultrafiltration using rhamnolipid: Flux decline, adsorption kinetics, and isotherm studies. J. Environ. Manag..

[8] Yaqub M., Lee S.H. (2019). Heavy metals removal from aqueous solution through micellar enhanced ultrafiltration: A review. Environ. Eng. Res..

[9] Camargo J.A., Alonso Á. (2006). Ecological and toxicological effects of inorganic nitrogen pollution in aquatic ecosystems: A global assessment. Environ. Int..

[10] Yenphan P., Chanachai A., Jiraratananon R. (2010). Experimental study on micellar-enhanced ultrafiltration (MEUF) of aqueous solution and wastewater containing lead ion with mixed surfactants. Desalination.

[11] Samper E., Rodríguez M., De la Rubia M.A., Prats D. (2009). Removal of metal ions at low concentration by micellar-enhanced ultrafiltration (MEUF) using sodium dodecyl sulfate (SDS) and linear alkylbenzene sulfonate (LAS). Sep. Purif. Technol..

[12] El Zeftawy M.A.M., Mulligan C.N. (2011). Use of rhamnolipid to remove heavy metals from wastewater by micellar-enhanced ultrafiltration (MEUF). Sep. Purif. Technol..

[13] Vibhandik A.D., Marathe K.V. (2014). Removal of Ni(II) ions from wastewater by micellar-enhanced ultrafiltration using mixed surfactants. Front. Chem. Sci. Eng..

[14] Fu Q., Zheng B., Zhao X., Wang L., Liu C. (2012). Ammonia pollution characteristics of centralized drinking water sources in China. J. Environ. Sci..

[15] Hilal N., Al-Zoubi H., Darwish N.A., Mohammad A.W., Abu Arabi M. (2004). A comprehensive review of nanofiltration membranes: Treatment, pretreatment, modeling, and atomic force microscopy. Desalination.

[16] Ghadge S., Chavan M., Divekar A., Vibhandik A., Pawar S., Marathe K. (2015). Mathematical Modelling for Removal of Mixture of Heavy Metal Ions from Wastewater Using Micellar Enhanced Ultrafiltration (MEUF) Process. Sep. Sci. Technol..

[17] Wang Z., Ma J., Tang C.Y., Kimura K., Wang Q., Han X. (2014). Membrane cleaning in membrane bioreactors: A review. J. Membr. Sci..

[18] Papp J.F., Lipin B.R. (2010). Kirk-Othmer Encyclopedia of Chemical Technology.

[19] Rahman P.K., Gakpe E. (2008). Production, Characterisation, and Applications of Biosurfactants-Review. Biotechnology.

[20] Ladewig B., Al-Shaeli M.N.Z. (2017). Fundamentals of Membrane Bioreactors, Materials.

[21] Moreno M., Mazur L.P., Weschenfelder S.E., Regis R.J., de Souza R.A.F., Marinho B.A., da Silva A., de Souza S.M.A.G.U., de Souza A.A.U. (2022). Water and wastewater treatment by micellar enhanced ultrafiltration—A critical review. J. Water Process Eng..

[22] Liu D.H., Lipták B.G. (1999). Groundwater and Surface Water Pollution.

[23] Kim B.K., Baek K., Yang J.W. (2004). Simultaneous removal of nitrate and phosphate using crossflow micellar-enhanced ultrafiltration (MEUF). Water Sci. Technol..

[24] Chen M., Jafvert C.T., Wu Y., Cao X., Hankins N.P. (2020). Inorganic anion removal using micellar enhanced ultrafiltration (MEUF), modeling anion distribution and suggested improvements of MEUF: A review. Chem. Eng. J..

[25] Sadr S.M.K., Saroj D.P. (2015). Membrane technologies for municipal wastewater treatment. Advances in Membrane Technologies for Water Treatment: Materials, Processes and Applications.

[26] Deriszadeh A. (2009). Improved MEUF Treatment of Produced Water Utilizing Naphthenic Acid Co-contaminants. Ph.D. Dissertation.

[27] Ferraz F.M., Povinelli J., Vieira E.M. (2013). Ammonia removal from landfill leachate by air stripping and absorption. Environ. Technol..

[28] Makkar R.S., Cameotra S.S. (1999). Biosurfactant production by microorganisms on unconventional carbon sources. J. Surfactants Deterg..

[29] Liu L., Luo X.B., Ding L., Luo S.L. (2018). Application of Nanotechnology in the Removal of Heavy Metal from Water. Nanomaterials for the Removal of Pollutants and Resource Reutilization.

[30] Miao L., Yang G., Tao T., Peng Y. (2019). Recent advances in nitrogen removal from landfill leachate using biological treatments—A review. J. Environ. Manag..

[31] Deng H., Huang Y. (2020). Chelating surfactant for the removal of heavy metals from wastewater and surfactant recovery. Desalin. Water Treat..

[32] Puasa S.W., Ruzitah M.S., Sharifah A.S.A.K. (2011). An overview of Micellar—Enhanced Ultrafiltration in Wastewater Treatment Process. Int. Conf. Environ. Ind. Innov..

[33] Zhao Y., Ren Y., Wang X., Xiao P., Tian E., Wang X., Li J. (2016). An initial study of EDTA complex based draw solutes in forward osmosis process. Desalination.

[34] Rahmati N.O., Pourafshari Chenar M., Namaghi H.A. (2017). Removal of free active chlorine from synthetic wastewater by MEUF process using polyethersulfone/titania nanocomposite membrane. Sep. Purif. Technol..

[35] Kang G.D., Cao Y. (2012). Development of antifouling reverse osmosis membranes for water treatment: A review. Water Res..

[36] Mulligan C.N., Yong R.N., Gibbs B.F. (2001). Surfactant-enhanced remediation of contaminated soil: A review. Eng. Geol..

[37] Mulligan C.N., Sharifi-Nistanak M. (2016). Conversion of sludge from a wastewater treatment plant to a fertilizer. Int. J. GEOMATE.

[38] Wang C., Tan J., Harle G., Gong H., Xia W., Zheng T., Yang D., Ge Y., Zhao Y. (2019). Ammonia Formation over Pd/Rh Three-Way Catalysts during Lean-to-Rich Fluctuations: The Effect of the Catalyst Aging, Exhaust Temperature, Lambda, and Duration in Rich Conditions. Environ. Sci. Technol..

[39] Robert M., Mercadé M.E., Bosch M.P., Parra J.L., Espuny M.J., Manresa M.A., Guinea J. (1989). Effect of the carbon source on biosurfactant production by *Pseudomonas aeruginosa* 44T1. Biotechnol. Lett..

[40] Valeri P., Morrone L.A., Romanelli L., Amico M.C., Piccinelli D. (1992). Effetti Dei Farmaci Antiinfiammatori Non Steroidei (Nsaids) Sulle Contrazioni Evocate Nell’Ileo Di Cavia. Ars Pharm..

[41] Mungray A.A., Kulkarni S.V., Mungray A.K. (2012). Removal of heavy metals from wastewater using micellar enhanced ultrafiltration technique: A review. Cent. Eur. J. Chem..

[42] Schwarze M. (2017). Micellar-enhanced ultrafiltration (MEUF)-state of the art. Environ. Sci. Water Res. Technol..

[43] Samal K., Das C., Mohanty K. (2017). Application of saponin biosurfactant and its recovery in the MEUF process for removal of methyl violet from wastewater. J. Environ. Manag..

[44] Silva SN R.L., Farias CB B., Rufino R.D., Luna J.M., Sarubbo L.A. (2010). Glycerol as substrate for the production of biosurfactant by *Pseudomonas aeruginosa* UCP0992. Colloids Surf. B Biointerfaces.

[45] Uysal A., Celik E. (2019). Removal of metals and recovery of released nutrients from municipal and industrial sludge using different biosurfactants. Desalin. Water Treat..

[46] Kurniawan T.A., Lo W.H., Chan G.Y.S. (2006). Physico-chemical treatments for removal of recalcitrant contaminants from landfill leachate. J. Hazard. Mater..

[47] Namaghi H.A., Mousavi S.M. (2014). Micellar-enhanced ultrafiltration of soft drink wastewater using anionic and mixed anionic/nonionic surfactants. J. Taiwan Inst. Chem. Eng..

[48] Fu H.-Y., Zhang Z.-B., Chai T., Huang G.-H., Yu S.-J., Liu Z., Gao P.-F. (2017). Study of the Removal of Aniline from Wastewater via MEUF Using Mixed Surfactants. Water.

